# Determinants of Quantitative Optical Coherence Tomography Angiography Metrics in Patients with Diabetes

**DOI:** 10.1038/s41598-017-02767-0

**Published:** 2017-05-31

**Authors:** Fang Yao Tang, Danny S. Ng, Alexander Lam, Fiona Luk, Raymond Wong, Carmen Chan, Shaheeda Mohamed, Angie Fong, Jerry Lok, Tiffany Tso, Frank Lai, Marten Brelen, Tien Y. Wong, Clement C. Tham, Carol Y. Cheung

**Affiliations:** 1 Department of Ophthalmology and Visual Sciences, The Chinese University of Hong Kong, Hong Kong, China; 2 Hong Kong Eye Hospital, Hong Kong, China; 3 Singapore Eye Research Institute, Singapore National Eye Center, Singapore, Singapore

## Abstract

Early microvascular damage in diabetes (e.g. capillary nonperfusion and ischemia) can now be assessed and quantified with optical coherence tomography-angiography (OCT-A). The morphology of vascular tissue is indeed affected by different factors; however, there is a paucity of data examining whether OCT-A metrics are influenced by ocular, systemic and demographic variables in subjects with diabetes. We conducted an observational cross-sectional study and included 434 eyes from 286 patients with diabetes. Foveal avascular zone (FAZ) area, FAZ circularity, total and parafoveal vessel density (VD), fractal dimension (FD), and vessel diameter index (VDI) from the superficial capillary plexus OCT-angiogram were measured by a customized automated image analysis program. We found that diabetic retinopathy (DR) severity was associated with increased FAZ area, decreased FAZ circularity, lower VD, lower FD, and increased VDI. Enlarged FAZ area was correlated with shorter axial length and thinner central subfield macular thickness. Decreased FAZ circularity was correlated with a reduction in visual function. Decreased VD was correlated with thinner macular ganglion-cell inner plexiform layer. Increased VDI was correlated with higher fasting glucose level. We concluded that the effects of ocular and systemic factors in diabetics should be taken into consideration when assessing microvascular alterations via OCT-A.

## Introduction

Diabetic retinopathy (DR), a common and specific microvascular complication of diabetes mellitus, is one of the leading global causes of preventable blindness^1^. Better stratification of individuals at high risk of vision-threatening complications of DR (e.g. diabetic macular edema [DME], and proliferative DR) who would benefit from early intervention is crucial for the prevention of vision loss. Microvascular damage in diabetes results in capillary nonperfusion and ischemia and upregulates angiogenic factors including vascular endothelial growth factor (VEGF). Microvascular damage stimulates pathologic neovascularization and increased vascular permeability, subsequently leading to the development of advanced stages of DR and DME^2^.

Optical coherence tomography-angiography (OCT-A), based on mapping erythrocyte movement over time by comparing sequential OCT B-scans (motion contrast) at a given cross-section, has been developed to provide depth-resolved visualization of the retinal microvasculature without intravenous dye injection^3,4^. A couple of publications have reported that microvascular changes (e.g. foveal avascular zone [FAZ] enlargement, microaneurysms, capillary dropout, neovascularization) can be detected in diabetic eyes using OCT-A with good agreement with fluorescein angiography^5–16^. Some of these studies have also measured FAZ size and vessel density and correlated with diabetic macular ischemia severity, demonstrating that the microvascular changes, and in particular, quantitative metrics derived from OCT-A have great potential to serve as biomarkers of DR. However, the morphology of vascular tissue is affected by different factors and there is a paucity of data examining whether OCT-A metrics are influenced by ocular, systemic and demographic variables in subjects with diabetes. Furthermore, there is also very few data on reliability of OCT-A measurement in subjects with diabetes. Such data are crucial before further investigating whether these OCT-A metrics are useful for assessing DR and its progression risk.

In this study, we first quantified the retinal microvasculature from OCT-angiograms generated by a swept-source based OCT-A device, in terms of FAZ area, FAZ circularity, vessel density (VD), fractal dimension (FD), and vessel diameter index (VDI) using a customized program and assessed its intra-session and inter-session reliability in patients with diabetes. Second, we examined the influences of a range of ocular (e.g. DR severity, axial length [AL], visual acuity [VA], macular thickness, subfoveal choroidal thickness), systemic (e.g. HbA_1c_, fasting glucose, blood pressure, body mass index [BMI], history of stroke) and demographic factors (e.g. age, duration of diabetes) on the OCT-A metrics in a cohort of patients with diabetes.

## Results

506 eyes from 301 subjects were eligible in this study. After quality check, we included 434 superficial capillary plexus OCT-angiograms from 286 subjects in the final analysis. Eyes were excluded due to low quality score (n = 26), motion artifacts (n = 12), blurry images (n = 24), signal loss (n = 7) and poor centration (n = 3). Table 1 shows the demographics between included eyes/subjects and excluded eyes/subjects. Among the included eyes, there were 171 eyes with no DR, 120 eyes with mild NPDR, 114 eyes with moderate NPDR, 25 eyes with severe NPDR and 4 eyes with PDR. Included subjects had diabetes for a mean of 13.5 (SD 9.37) years. 55 eyes (from 12 mild, 34 moderate, 8 severe NPDR and 1 PDR) had DME.

**Table 1:** Clinical Characteristics of included diabetic participants in the analysis compared with excluded participants.

	Included	Excluded	P-value
	by eyes (n = 434)	by eyes (n = 72)	
Diabetic retinopathy severity (no/mild/moderate/severe or above)	171/120/114/29 (39%/28%/26%/7%)	30/11/13/18 (41%/16%/18%/25%)	**<0.001**
Presence of diabetic macular edema	55 (12.7%)	10 (13.9%)	0.775
Axial length, mm	23.89 (1.29)	24.38 (1.57)	**0.023**
Anterior chamber depth, mm	3.29 (0.65)	3.32 (0.52)	0.641
Spherical equivalent, diopter	−0.95 (2.46)	−2.60 (5.11)	**0.015**
Central corneal thickness, µm	557.31 (32.87)	545.41 (46.61)	0.070
Intraocular pressure, mmHg	15.98 (3.01)	17.00 (3.26)	**0.022**
LogMAR	0.21 (0.16)	0.38 (0.30)	<**0.001**
Ocular perfusion pressure, mmHg	55.70 (9.27)	56.89 (8.21)	0.277
Central subfield macular thickness, µm	256.0 (36.99)	292.36 (109.94)	**0.023**
Average ganglion cell inner plexiform layer thickness, µm	79.2 (10.17)	76.06 (11.22)	**0.043**
Average peripapillary retinal nerve fiber layer thickness, µm	91.33 (10.78)	88.88 (9.62)	0.100
Subfoveal choroidal thickness, µm	194.76 (78.61)	176.26 (91.32)	0.161
	**by subjects (n = 286)**	**by subjects (n = 15)**	
Gender, Female	164 (57.3%)	10 (66.7%)	0.476
Age, year	65.69 (10.73)	67.07 (7.89)	0.530
Duration of diabetes, year	13.52 (9.37)	8.69 (7.81)	**0.035**
Body mass index, kg/m^2^	26.18 (5.07)	24.91 (3.62)	0.217
Systolic blood pressure, mmHg	144.22 (19.81)	143.83 (13.57)	0.919
Diastolic blood pressure, mmHg	77.79 (10.41)	76.27 (10.99)	0.191
Pulse pressure, mmHg	67.2 (17.9)	73.9 (13.7)	0.609
Mean arterial blood pressure, mmHg	99.93 (11.21)	98.79 (9.78)	0.669
HbA_1c_, %	7.42 (1.37)	7.21 (1.18)	0.518
Fasting glucose, mmol/L	7.82 (2.30)	6.77 (2.05)	0.128
Creatinine, µmol/L	98.53 (83.18)	96.83 (43.54)	0.903
Estimated glomerular filtration rate, mL/min/1.73 m^2^	72.41 (23.05)	63.25 (19.52)	0.141
Total cholesterol, mmol/L	4.18 (0.91)	4.06 (0.45)	0.382
HDL cholesterol, mmol/L	1.31 (0.41)	1.26 (0.33)	0.642
LDL cholesterol, mmol/L	2.26 (0.69)	2.01 (0.34)	**0.037**

Data are N (%) or mean (standard deviation).

HDL = High-density lipoprotein; LDL = Low-density lipoprotein; LogMAR: logarithm of the minimum angle of resolution.

Table 2 shows the reliability analysis for the OCT-A measurements using our customized program in the subset of 30 diabetic subjects. Intra-session repeatability of the OCT-A metrics were high, with ICCs ranged 0.989 to 0.633 while the inter-session reproducibility was slightly lower but the overall performance was still good, with ICCs ranged 0.987 to 0.624. A Bland-Altman plot of FAZ measurement between fluorescein angiography (FA) and OCT-A is showed in the Supplement (Supplemental material 1). The mean difference was −0.01 mm^2^ with 95% limits of agreement between −0.07 and 0.05 mm^2^, and the ICC between two measurements was 0.983 (95% CI 0.965 to 0.991).

**Table 2:** Reliability estimates of quantitative optical coherence tomography-angiography (OCT-A) metrics measurements using an automated analysis program in a subset of 30 eyes from 30 diabetic participants. Two OCT-A scans were acquired during first visit for these participants for intra-session repeatability assessment. These 30 patients were also invited for another visit for OCT-A scan within 2 weeks for inter-session reproducibility assessment.

	Intra-section		Inter-section	
	ICC (95% CI)	CR (95% CI)	ICC (95% CI)	CR (95% CI)
FAZ Area (mm^2^)	0.976 (0.950–0.988)	0.040 (0.036–0.043)	0.967 (0.932–0.984)	0.050 (0.045–0.055)
FAZ Circularity	0.751 (0.540–0.873)	0.125 (0.114–0.138)	0.720 (0.490–0.856)	0.129 (0.117–0.141)
Total Vessel Density (%)	0.840 (0.691–0.920)	0.069 (0.063–0.076)	0.777 (0.583–0.887)	0.090 (0.081–0.098)
Parafoveal Vessel Density (%)	0.826 (0.667–0.913)	0.078 (0.071–0.085)	0.774 (0.577–0.885)	0.098 (0.088–0.107)
Fractal Dimension	0.989 (0.978–0.995)	0.010 (0.009–0.011)	0.987 (0.972–0.994)	0.010 (0.009–0.011)
Vessel Density Index (mm)	0.633 (0.358–0.807)	0.001 (0.0008–0.001)	0.624 (0.346–0.802)	0.001 (0.0008–0.001)

CR: Coefficient of repeatability; FAZ: Foveal avascular zone; ICC: Intra-class correlation coefficient.

Univariate analyses between ocular (DR severity, presence of DME, logMAR, AL, ACD, SE, CCT, IOP, OPP, central subfield and average macular thicknesses, average GC-IPL thicknesses, peripaillary RNFL thickness, subfoveal choroidal thickness), systemic (fasting glucose, HbA_1c_, lipids, eGFR, blood pressure, BMI, smoking, anti-hypertensive and anti-dyslipidemic medication, history of stroke, heart disease, arrhythmia and renal disease) and demographic (age, gender, duration of diabetes) variables with OCT-A metrics are summarized in the Supplement (Supplemental material 2).

**Figure 1:**
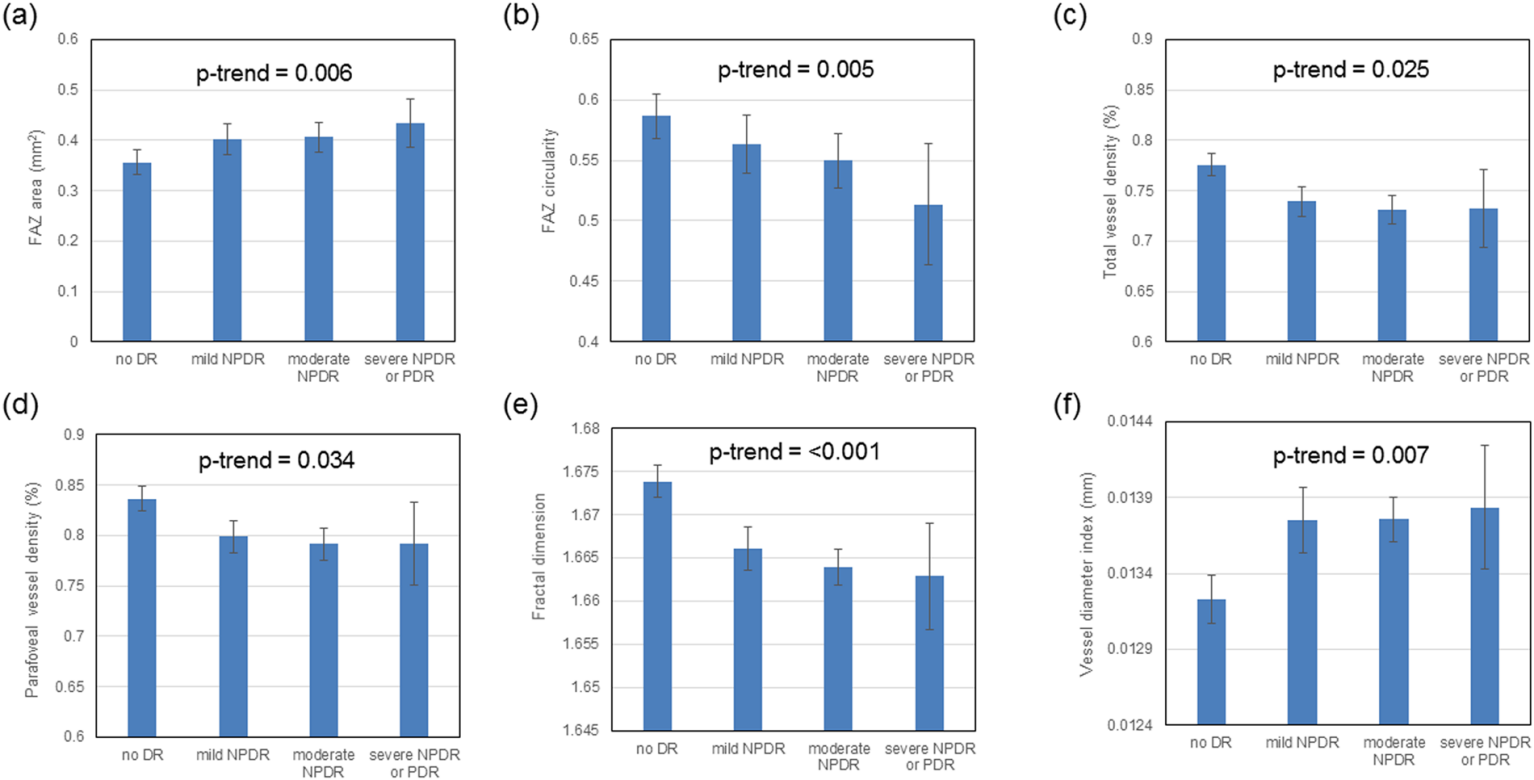
Associations between (**a**) foveal avascular zone (FAZ) area, (**b**) FAZ circularity, (**c**) total vessel density, (**d**) parafoveal vessel density, (**e**) fractal dimension, and (**f**) vessel diameter index with diabetic retinopathy severity. P-trend represents the trend across four increased DR severity categories. Vertical bars indicate 95% confidence intervals.

Table 3 shows the multiple regression models of (a) FAZ area, (b) FAZ circularity, (c) total VD, (d) parafoveal VD, (e) FD, and (f) VDI (dependent variables) with variables that showed significant associations in univariate analyses (independent variables). In general, all OCT-A metrics were significantly associated with DR severity but not presence of DME. Enlarged FAZ area was significantly correlated with shorter axial length, and thinner central subfield macular thickness. Decreased FAZ circularity was significantly correlated with a reduction in VA. Decreased total and parafoveal VD were significantly correlated with thinner GC-IPL thickness. Increased VDI was correlated with higher fasting glucose level. OCT-A metrics were not associated with age, duration of diabetes, CCT, OPP, subfoveal choroidal thickness, and other systemic factors (e.g. blood pressure, lipids, eGFR and BMI). Figure 1 shows the associations between (a) FAZ area, (b) FAZ circularity, (c) total VD, (d) parafoveal VD, (e) FD, and (f) VDI with DR severity.

**Table 3:** Multiple regression models of (a) foveal avascular zone (FAZ) area, (b) FAZ circularity, (c) total vessel density, (d) parafoveal vessel density and (e) fractal dimension (f) vessel diameter index (dependent variables) with variables that showed significant associations in univariate analyses (independent variables).

	Beta coefficient	SE	95% CI	P-value
**(a) FAZ area (mm** ^**2**^ **)**				
Severe or above DR vs. no DR	0.100	0.153	0.042 to 0.153	**0.001**
Moderate DR vs. no DR	0.068	0.030	0.032 to 0.104	**<0.001**
Mild DR vs. no DR	0.054	0.018	0.015 to 0.092	**0.007**
Axial length, mm	−0.017	0.007	−0.029 to −0.004	**0.010**
Central subfield macular thickness, µm	−0.001	3.00 × 10^−4^	−0.002 to −3.00 × 10^−4^	**0.005**
Subfoveal choroidal thickness, µm	1.00 × 10^−4^	7.25 × 10^−5^	−1.63 × 10^−5^ to 2.68 × 10^−4^	0.083
Gender (male vs. female)	−0.030	0.165	−0.062 to 0.003	0.072
**(b) FAZ circularity**				
Severe or above DR vs. no DR	−0.054	0.292	−0.111 to 0.003	0.064
Moderate DR vs. no DR	−0.036	0.152	−0.066 to −0.007	**0.016**
Mild DR vs. no DR	−0.036	0.151	−0.062 to 0.002	**0.034**
LogMAR, per unit	−0.079	0.038	−0.152 to −0.005	**0.036**
Average macular thickness, µm	−0.001	3.56 × 10^−4^	−0.001 to 3.72 × 10^−5^	0.064
History of stroke (yes vs. no)	−0.044	0.023	−0.088 to 1.97 × 10^−4^	0.051
**(c) Total vessel density (%)**				
Severe or above DR vs. no DR	−0.051	0.023	−0.097 to −0.005	**0.029**
Moderate DR vs. no DR	−0.043	0.009	−0.061 to −0.025	**<0.001**
Mild DR vs. no DR	−0.032	0.009	−0.050 to −0.014	**0.001**
Average GC-IPL, µm	0.001	4.00 × 10^−4^	5.64 × 10^−5^ to 0.002	**0.035**
Total cholesterol, mmol/L	0.006	0.006	−0.006 to 0.019	0.301
LDL cholesterol, mmol/L	0.005	0.008	−0.011 to 0.022	0.508
**(d) Parafoveal vessel density (%)**				
Severe or above DR vs. no DR	−0.052	0.025	−0.101 to −0.003	**0.039**
Moderate DR vs. no DR	−0.043	0.010	−0.063 to −0.024	**<0.001**
Mild DR vs. no DR	−0.032	0.010	−0.052 to −0.031	**0.001**
Average GC-IPL, µm	0.001	4.00 × 10^−4^	1.88 × 10^−4^ to 0.002	**0.016**
Total cholesterol, mmol/L	0.005	0.007	−0.008 to 0.018	0.439
LDL cholesterol, mmol/L	0.006	0.009	−0.008 to 0.018	0.481
History of arrhythmia (yes vs. no)	0.002	0.014	−0.025 to 00.28	0.904
**(e) Fractal dimension**				
Severe or above DR vs. no DR	−0.010	0.003	−0.016 to −0.003	**0.003**
Moderate DR vs. no DR	−0.009	0.002	−0.012 to −0.006	**<0.001**
Mild DR vs. no DR	−0.006	0.002	−0.010 to −0.003	**<0.001**
Subfoveal choroidal thickness, µm	−1.52 × 10^−5^	8.11 × 10^−6^	−3.11 × 10^−5^ to 7.21 × 10^−7^	0.061
HbA_1c_, %	−0.001	0.001	−0.002 to 4.27 × 10^−4^	0.266
History of Renal disease (yes vs. no)	0.004	0.002	−0.001 to 0.009	0.086
Current smoking (yes vs. no)	−0.003	0.002	−0.00λo 0.001	0.095
**(f) Vessel Diameter Index (mm)**				
Severe or above DR vs. no DR	0.001	2.18 × 10^−4^	2.87 × 10^−4^ to 0.001	**0.001**
Moderate DR vs. no DR	0.001	1.31 × 10^−4^	3.11 × 10^−4^ to 0.001	**<0.001**
Mild DR vs. no DR	4.42 × 10^−4^	1.71 × 10^−4^	1.07 × 10^−4^ to 0.001	**0.01**
Diastolic blood pressure, mmHg	−1.13 × 10^−5^	6.00 × 10^−6^	−2.31 × 10^−5^ to 4.51 × 10^−7^	0.059
Fasting glucose, mmol/L	6.14 × 10^−5^	2.58 × 10^−5^	1.09 × 10^−5^ to 1.12 × 10^−4^	**0.017**

DR: diabetic retinopathy; FAZ: Foveal avascular zone; GC-IPL: Ganglion cell inner plexiform layer; LogMAR: logarithm of the minimum angle of resolution; SE: standard error.

## Discussion

In this study, we showed that morphology of the superficial capillary network at the macula, as examined via OCT-A, can be reliably measured with the automated analysis program, and were significantly and independently associated with DR severity, AL, VA, GC-IPL, and fasting glucose level in patients with diabetes.

Knowing the range of ocular, systemic and demographic parameters that may affect OCT-A measurements in patients with diabetes is crucial before OCT-A can be considered for serving as biomarker of DR. Among the determinants of OCT-A metrics, we showed that DR severity had the most impact and was associated with increased FAZ area, decreased FAZ circularity, lower VD, lower FD and increased VDI, suggesting the extent of microvascular damage (e.g. macular ischemia) can be quantified from OCT-A. Our findings were consistent with previous studies with smaller sample size^13,16–18^, and were in concordance with FA evaluation, in which macular ischemia, indicated by capillary nonperfusion, is associated with development and progression of DR and DME^19^. Early microvascular changes in diabetic eyes may be visualized using OCT-A potentially before detection by clinical examination^11^. Our cross-sectional results hold implications that OCT-A may identify diabetic individuals at risk of developing DR progression. This may have important clinical significance as patients at higher risk can benefit from more intensive interventions (e.g. intensive glycemic control, medical therapies) to reduce the risk of vision loss due to diabetes at an early stage.

The FAZ, containing metabolically active photoreceptors, is responsible for central vision. Previous studies showed that diabetic eyes are more likely to have FAZ enlargement compared with healthy control eyes, consistent with the observation based on FA^8,11,16,20,21^. Recent OCT-A studies have further found that enlarged FAZ is correlated with reduced VA^8,17,22^. Nevertheless, it is noted that size of FAZ varies among individuals and thus it is not easy to assess possible pathologic FAZ enlargement in the setting of retinal diseases^23,24^. Furthermore, AL had a significant influence on FAZ area measurement as shown in our study which is possibly due to ocular magnification, similar to other OCT measurements^25,26^. Thus, FAZ area may not be a sensitive marker to assess foveal health and relate to central vision. In addition to measurement of FAZ area, we defined a new dimensionless parameter presented as a simple scale from 0.00 to 1.00 to quantify FAZ circularity as proposed by Domalpally *et al*. to assess progression of geographic atrophy^27^. We found that lower value of FAZ circularity index, indicating a more irregular shape of FAZ, was significantly correlated with poorer VA. Reduction in perfusion may lead to ischemia in the fovea which has a significant impact on VA in DR^28^. The FAZ circularity index may allow quantifying disruption of the terminal capillary ring at the fovea and it may be a better measure for assessing the degree of microvascular damage at FAZ which is more related to vision. Further longitudinal studies are warranted to examine the predictive value of FAZ circularity for VA in patients with diabetes.

Consistent with previous reports in normal eyes, we also found that an inverse association between FAZ area on the superficially capillary plexus and central subfield macular thickness, suggesting that a thinner foveal thickness is characterized by less compact and a larger degree of separation of the inner retinal layers over the foveolar center, and hence a larger FAZ^24,29,30^. We did not observe any statistically significant associations between OCT-A metrics and the presence of DME on the superficially capillary plexus. Lee *et al*. recently demonstrated that eyes with DME have enlarged FAZ area and lower vascular density only at deep capillary plexus, but not superficial capillary plexus, compared with eyes without DME^31^. They further found that DME eyes with a poor response to anti-VEGF agents have greater damage at the deep capillary plexus, but not superficial plexus, compared with DME eyes with good response^31^. Therefore, our finding might be due to microvascular alternations occurring at the deep capillary plexus, not at the superficial plexus during the pathogenesis of DME. In this study, we only focused to analyze OCT-angiograms of the superficial capillary plexus as the shadowgraphic projection artifacts from superficial capillary plexus are common and interfere with the visualization of deeper capillary plexus^32^. The current OCT-angiograms of the deep capillary plexus did not reflect the erythrocyte movement within deep capillary network. The morphology of deep capillary plexus can only be accurately assessed or measured after projection artifacts are effectively resolved. New algorithms to remove this artifact are currently in development^33,34^. We plan to further analyze the deep capillary plexus in this cohort, once the projection artifact issue on OCT-angiograms of deep capillary plexus is resolved. Furthermore, DME is usually caused by the breakdown of the blood–retinal barrier and the leakage of intraretinal fluid. However, it is noted that OCT-A does not provide information on blood-retina barrier integrity as OCT-A cannot depict leakage.

We observed a positive correlation between VD on the superficially capillary plexus and GC-IPL in our diabetic cohort. It is increasingly shown that retinal neurodegeneration may precede signs of microvasculopathy in diabetes^35^. Previous clinical studies also reported that GC-IPL and RNFL thinning, indicating retinal neurodegeneration, are associated with DR^36,37^. It has been suggested that the retinal dysfunction associated with diabetes may be viewed as a change in the retinal neurovascular unit^38^. OCT-A may facilitate study of the link between microangiopathy and neurodegeneration in DR as it can allow simultaneous visualization of microvascular abnormalities and retinal layer structure from the human eye. Nonetheless, a clear relationship between capillary network and retinal neurodegeneration in diabetes needs to be further investigated.

In regard to systemic factors, we found that increased VDI, indicating widening of retinal capillaries, was associated with increased glucose level, in addition to increased DR severity level. In line with our data, it is previously showed that widened retinal vascular caliber as measured from fundus photography quantitatively is associated with increased glucose level and DR severity^39,40^. The possible pathophysiological processes underlying the association is that hyperglycemia and hypoxia initiate retinal vasodilation, leading to hyperperfusion, while hyperperfusion in turn interferes with autoregulation, resulting in further vasodilation^41^. It has been suggested that measurement of retinal vascular widening can be a potential biomarker to predict early microvascular complications in diabetes^42,43^. Other systemic variables (e.g. blood pressure, lipids) and demographic factors (e.g. age and duration of diabetes) did not influence OCT-A metrics in this study cohort.

Quantification of capillary network from OCT-A to identify early and subtle microvascular changes objectively in diabetic eyes to optimize patient management is one of the next important milestones for the application of OCT-A into clinical practice. Different groups have started to develop automated and semi-automated algorithms to quantify the capillary network from OCT-A for other OCT devices^13,44–46^. Currently, there is no available build-in software for quantitative OCT-A measurement for the Triton DRI OCT. Therefore, we customized an automated MATLAB program for image analysis which reduced manual intervention and minimized measurement variability. We first applied noise reduction by a non-local means denoising filter in our image processing algorithm in order to reduce the noise, sharpen the objects and improve signal-to-noise ratio while preserving the image features even for low contrast regions. Our program applied local threshold for binarization, instead of setting a global threshold, so the details in the part of the image with lower signal-to-noise ratio will not be missed. We showed that the intra-session repeatability and inter-session reproducibility of OCT-A metrics obtained from Triton DRI OCT were generally good, suggesting that our algorithm, accounting for signal-to-noise ratio variability, can provide a reliable, objective and automated approach to evaluate macular capillary nonperfusion and morphology.

Our study had several strengths, including prospective study design, standardized image acquisition protocol and strict definition of image artifacts for quality control in reading centre, and a wide range of systemic and ocular factors. This study also had several limitations. First, like other studies, the causal relationships between OCT-A metrics and the factors were unclear, due to the cross-sectional nature of our data. Second, we only evaluated the superficial capillary plexus with a 3 mm × 3 mm field of view centered at fovea and we did not evaluate the peripheral retina. Third, image artifacts are very common in OCT-A as it is extremely sensitive to motion. We had to exclude a certain number of eyes with image artifacts (14.2%), which may have introduced selection bias and limited the generalizability of results. Nevertheless, we believe this will not affect our interpretation of the results significantly as the sample size of the included eyes is large (n = 434). Fourth, our program currently cannot distinguish nonperfused area and area of cystoid edema from the dark area of OCT-angiograms. A method that can segment cystoid edema and integrate into the angiographic data is needed^47^. Furthermore, it is noted that some results of the study should be seen as exploratory ones, and confirmatory studies are necessary to evaluate the findings that were considered statistically significant.

In summary, the effects of ocular and systemic factors in patients with diabetes should be taken into consideration when assessing microvascular alterations at macula via OCT-A. Future research directions will be focused on evaluating OCT-A metrics as surrogate markers of foveal health to assess vision-threatening DR risk as well as its applications for predicting disease onset, progression, and prognosis.

## Methods

### Subjects

We included subjects from an ongoing prospective, observational clinical study. Inclusion criteria comprised of patients with type 1 or type 2 diabetes mellitus, treatment naïve, spherical refractive error within the range of −8.5 to + 4.0 diopter (D) with less than 5.0 D of cylinder, visual acuity (VA) of not worse than Snellen 20/200. Dilated biomicroscopic fundus examination was performed for assessing DR severity and DME by retinal specialists before OCT-A imaging, according to the International Clinical Diabetic Retinopathy and Diabetic Macular Edema Disease Severity Scales^48^. DR severity was categorised into absence of DR, mild, moderate and severe nonproliferative DR (NPDR), or proliferative diabetic retinopathy (PDR). This study was conducted in accordance with the 1964 Declaration of Helsinki and was approved by the Kowloon Central/East Research ethics committee. Written informed consent was obtained from all subjects.

In the current study, participants were consecutively recruited from July 2015 through December 2016 from CUHK Eye Centre, Hong Kong Eye Hospital. Exclusion criteria for study eyes included: 1) prior retinal surgery, intravitreal injection, macular laser photocoagulation or pan-retinal laser photocoagulation; 2) eye pathology that interferes with imaging (e.g. dense cataract, corneal ulcer); 3) glaucoma and 4) presence of epiretinal membrane and other maculopathy not related to diabetes (e.g. wet age-related macular degeneration).

### OCT-A Imaging

All the diabetic patients underwent OCT-A with a prototype swept-source OCT (Triton DRI-OCT, Topcon, Inc, Tokyo, Japan), which contains a swept-source with a wavelength of 1,050 nm light source and a speed of 100,000 A-scans per second. Volumetric OCT scans centered on the fovea were obtained with a scan area of 3 mm × 3 mm containing 320 × 320 A-scans. We used the latest built-in software (IMAGEnet6) to generate OCT-angiograms which can provide improved detection sensitivity of low blood flow and reduced motion artifacts without compromising axial resolution^49^. An image quality score ranged from 0 to 100 was given by the software for each volumetric OCT scan. The software is able to separately detect four horizontal depth-resolved slabs - the superficial capillary plexus, the deep capillary plexus, the outer retina, and the choriocapillaris, providing depth-resolved visualization of the retinal and choroid vasculature.

Although the built-in software of Triton can generate two retinal capillary plexuses. In the current analysis, we only focus on the superficial capillary plexus (en face slab from the internal limiting membrane to the inner border of the inner nuclear layer), as shadowgraphic projection artifacts from superficial capillary plexus on the deep capillary plexus cannot be resolved at this stage and may confound the results^50^.

### OCT-A Quality Control

A single reader (FYT) carefully evaluated each OCT-angiogram and OCT cross-sectional B-scan images in the CUHK Ocular Reading Centre before the quantitative analysis. The reader was masked to all patient characteristics including DR and DME severity. OCT-angiograms with significant image artifacts and poor image quality were excluded from analysis, including 1) quality score of below 40; 2) motion artifacts (e.g. vessel discontinuity or significant residual motion lines); 3) inaccurate segmentation of tissue layers or slabs; 4) blurry images (e.g. due to media opacity or axial movement); 5) signal loss (e.g. due to eye blinking) or 6) poor centration (i.e. fovea not at center).

### Quantification of superficial capillary network

En-face images of the OCT-angiograms are exported in grayscale from the built-in software. The images are then imported into an automated customized MATLAB program for image analysis. First, a non-local means (NLM) denoising filter^51^ was applied to the grayscale images in order to reduce the background noise, sharpen the blood vessels and improve signal-to-noise ratio while preserving the image features. The NLM filter is a non-linear neighbourhood filter in which the pixel/voxel value to be restored is replaced by a weighted average of the pixel intensities in the entire noisy image. The denoised image was then binarized using phansalkar adaptive local thresholding method with white pixels representing the blood vessels and black pixels representing the background. Region growing method was then used to identify the FAZ starting from a seed point.

The area of the FAZ (mm^2^) was calculated by counting total numbers of pixels within the region in scale. The contour of FAZ was further delineated. The length of the contour was measured based on pixel-to-pixel distance in scale and defined as the perimeter of the FAZ. Using this value, a circle with the same perimeter was defined with 2πr and was projected into a pixel matrix with same scale as the OCT-A images. The theoretical area occupied by the circle was measured based on the total number of pixels covered by the circle in the pixel matrix. The FAZ circularity index was calculated as the ratio of measured area of FAZ to the projected area of the circle, with a range of 0.0 to 1.0. A ratio closer to 0.0 indicates an irregular shape of FAZ, and a ratio closer to 1.0 indicates a circular shape of FAZ^27^. Total and parafoveal vessel density (VD) (%) were calculated based on the Early Treatment Diabetic Retinopathy Study (ETDRS) grid (1, 3 mm). The parafoveal region was defined as an annulus with an outer diameter of 3 mm and an inner diameter of 1 mm. Non-perfused regions were defined as dark areas from the binarized image larger than 0.02 mm^2^ ^44^. VD was calculated as the percentage of area not defined as non-perfusion regions over total area within the interested region^13^. The binarized image was also skeletonized^52^ and fractal dimension (FD) was then calculated by using box-counting method. Vessel diameter index (VDI) (mm) was calculated as the area occupied by blood vessel from the binarized image over the total length of blood vessel from the skeletonized image^13^.

**Figure 2:**
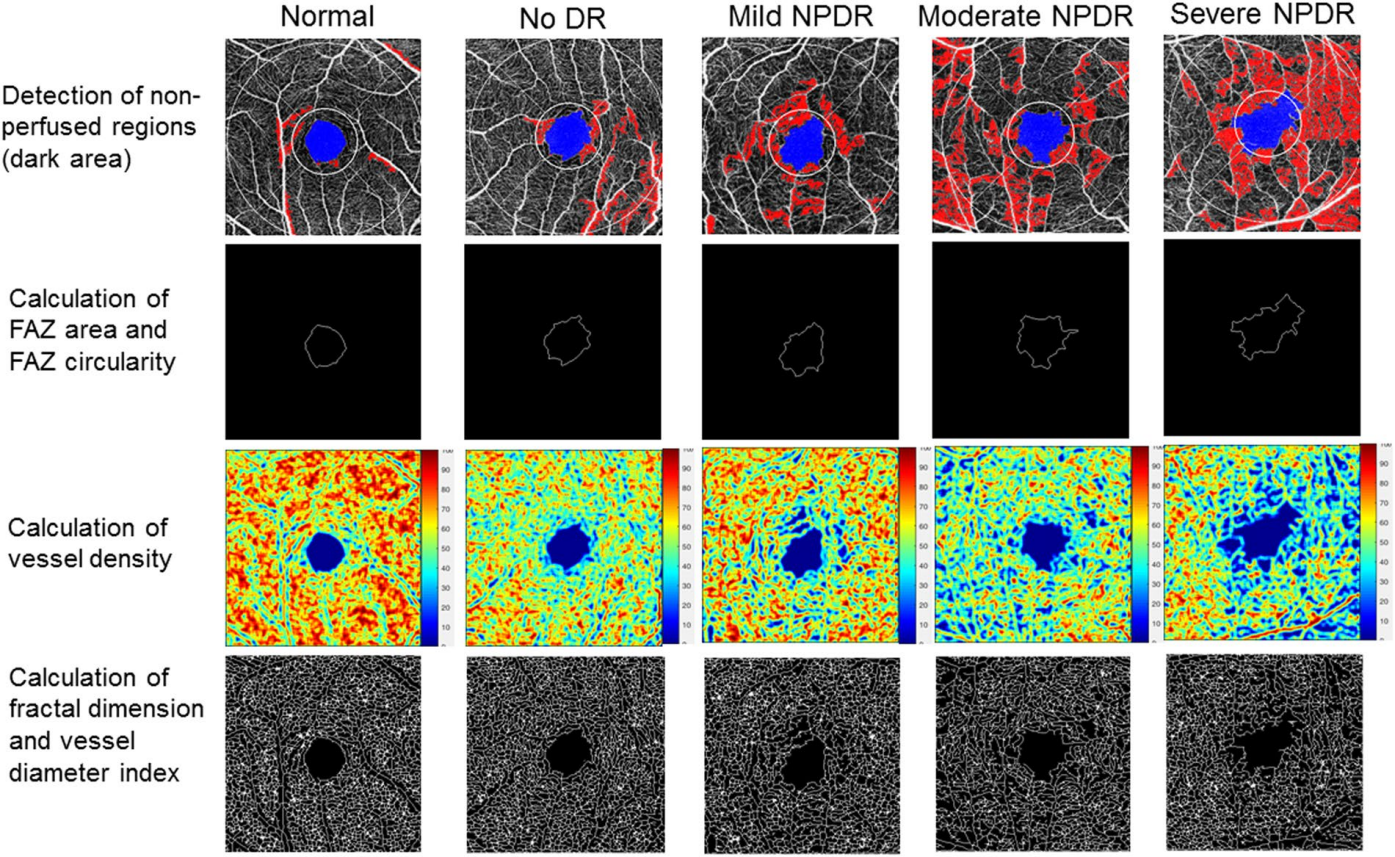
Quantification of retinal capillary network from OCT-angiograms using our customized program in normal and patients with different DR severity. A series of OCT-A metrics including foveal avascular zone (FAZ) area, FAZ circularity, total and parafoveal vessel density, fractal dimension and vessel diameter index can be calculated automatically. The parafoveal region was defined as an annulus with an outer diameter of 3 mm and an inner diameter of 1 mm.

Figure 2 demonstrates capillary network can be quantitatively measured from OCT-angiograms using our customized program in patients with different DR severity.

### Reliability Assessment

Two consecutive OCT-A scans were performed on one randomly selected eye from a subset of 30 diabetic subjects (12 eyes with no DR, 7 eyes with mild NPDR, 8 eyes with moderate NPDR, 3 eyes with severe NPDR) during the study for intra-session repeatability assessment. Each subject was instructed to sit back between the two scans while the OCT light beam was realigned. These 30 patients were also invited for another visit for OCT-A scan within 2 weeks for inter-session reproducibility assessment. Both repeated scans were not registered with the first scan. In addition, fluorescein angiography (FA) was performed in a subset of 17 subjects (33 eyes; 7 no DR, 8 mild NPDR, 16 moderate NPDR, 5 sever NPDR) with Spectralis HRA+OCT device (Heidelberg Engineering,  Heidelberg, Gemany). FAZ area was manually measured from the early phase FA image in each eye for agreement assessment.

### Measurement of ocular factors

Best-corrected visual acuity (VA) was measured using Snellen charts and then converted to logarithm of the minimum angle of resolution (logMAR) VA for the purposes of statistical analysis. Intraocular pressure (IOP) and central corneal thickness (CCT) were measured with a noncontact tono/pachymeter (TONOPACHY™ 530P, Nidek Co., Ltd., Japan) before pupil dilation. Ocular perfusion pressure (OPP) was calculated by subtracting the IOP from the 2/3 of the mean arterial blood pressure. The static refraction of each eye was measured using an autorefractor (ARK-510A, Nidek Co., Ltd., Japan) and the spherical equivalent (SE) was reported. Axial length (AL) was measured with a non-contact partial coherence laser interferometry (IOL Master, Carl Zeiss Meditec, Dublin, US). The mean of five measurements was used in the analysis. Central subfield and average macular thicknesses, average ganglion cell-inner plexiform layer (GC-IPL) thicknesses and average peripapillary retinal nerve fiber layer (RNFL) thickness were obtained with Cirrus HD-OCT (Carl Zeiss Meditec Inc., Dublin, CA, USA). Subfoveal choroidal thickness was obtained from horizontal scan with the Triton SS-OCT device, measured by the built-in calliper.

### Measurement of systemic factors

Each patient’s medical record was reviewed for the most recent fasting blood tests including serum total cholesterol, high-density lipoprotein (HDL) cholesterol, low-density lipoprotein (LDL) cholesterol, glycosylated hemoglobin (HbA_1c_), creatinine and glucose levels. Estimated glomerular filtration rate (eGFR) is derived from serum creatinine using the recently developed Chronic Kidney Disease Epidemiology Collaboration equation. Duration of diabetes, history of associated systemic diseases (e.g. hypertension, hypercholesterolemia, arrhythmia) and other diabetic complications (e.g. stroke, myocardial infarction, kidney diseases) were elicited from interviewed-based questionnaires, and the information was further checked and confirmed from each patients’ medical record by physicians. Systolic and diastolic blood pressures were measured using a digital automatic blood pressure monitor (model Avant 2120; Nonin Medical, Inc., Plymouth, MN, USA), after the subject was seated for at least 5 minutes. Pulse pressure was calculated as systolic blood pressure minus diastolic blood pressure. Current smoking was defined as those currently smoking any number of cigarettes (i.e. current vs. past/never). Body mass index (BMI) was calculated as body weight (in kilograms) divided by body height (in meters), squared.

### Statistical Analysis

All statistical analyses were performed using IBM SPSS statistics version 22.0. Normality of the variables was examined using histograms. OCT-A metrics (FAZ area, FAZ circularity, total VD, parafoveal VD, FD, VDI) were analyzed as continuous variables. Intra-class correlation coefficient (ICC) and the coefficient of repeatability (CR) were calculated to assess the intra-session repeatability and inter-session reproducibility. With a sample size of 30, we can detect an ICC of 0.86 with a 95% confidence interval width of 0.2 when two raters are used^53^. Bland-Altman plot was created to assess the agreement of FAZ area measurement between FA and OCT-A. Generalized estimating equations (GEE) models were performed to determine ocular, systemic and demographic factors (independent variables) associated with OCT-A metrics (dependent variables), adjusting for inter-eye correlation. Factors significant at p < 0.05 with OCT-A metrics from univariable analyses were included in multiple analyses.

## Electronic supplementary material

Supplementary information
