# Author Correction: Determinants of Quantitative Optical Coherence Tomography Angiography Metrics in Patients with Diabetes

**DOI:** 10.1038/s41598-018-25619-x

**Published:** 2018-05-04

**Authors:** Fang Yao Tang, Danny S. Ng, Alexander Lam, Fiona Luk, Raymond Wong, Carmen Chan, Shaheeda Mohamed, Angie Fong, Jerry Lok, Tiffany Tso, Frank Lai, Marten Brelen, Tien Y. Wong, Clement C. Tham, Carol Y. Cheung

**Affiliations:** 1 Department of Ophthalmology and Visual Sciences, The Chinese University of Hong Kong, Hong Kong, China; 2 Hong Kong Eye Hospital, Hong Kong, China; 3 Singapore Eye Research Institute, Singapore National Eye Center, Singapore, Singapore

Correction to: *Scientific Reports* 10.1038/s41598-017-02767-0, published online 31 May 2017

The original version of this Article contained a typographical error in the spelling of the author Clement C. Tham, which was incorrectly given as Clement C. C. Tham. This has now been corrected in the PDF and HTML versions of the Article, and in the accompanying Supplementary Information file.

