# Supplementary material for: Determinants of Quantitative Optical Coherence Tomography Angiography Metrics in Patients with Diabetes
Source: Sci Rep. 2017 May 31;7:2575. doi: 10.1038/s41598-017-02767-0 (PMC5451475; doi:10.1038/s41598-017-02767-0)
Supplement: Supplementary file 1 — Supplementary information [file 41598_2017_2767_MOESM1_ESM.doc]

**Supplementary information**

**Determinants of Quantitative Optical Coherence Tomography Angiography Metrics in Patients with Diabetes**

Fang Yao Tang, MD^1^, Danny S. Ng, MD, ^1^, Alexander Lam, PhD^1^, Fiona Luk, MD^1,2^, Raymond Wong, MD^1,2^, Carmen Chan, MD^1,2^, Shaheeda Mohamed, MD^1,2^, Angie Fong, MD^1,2^, Jerry Lok, MD^1,2^, Tiffany Tso, BSc^1^, Frank Lai, MD^1^, Marten Brelen, MD^1^, Tien Y. Wong, MD, PhD^3^, Clement C. Tham, MD^1,2^, Carol Y. Cheung, PhD^1^

1. Department of Ophthalmology and Visual Sciences, The Chinese University of Hong Kong, Hong Kong

2. Hong Kong Eye Hospital, Hong Kong

3. Singapore Eye Research Institute, Singapore National Eye Center, Singapore

Correspondence to: Carol Y. Cheung

Email: carolcheung@cuhk.edu.hk

Address: CUHK Eye Centre, Hong Kong Eye Hospital, 147K Argyle Street, Kln, Hong Kong

T: +852 3943 5831 / F: +852 2715 9490

**Figure.** Bland-Altman plot of the agreement of foveal avascular zone (FAZ) area measurement between fluorescein angiography (FA) and optical coherence tomography angiography (OCT-A).


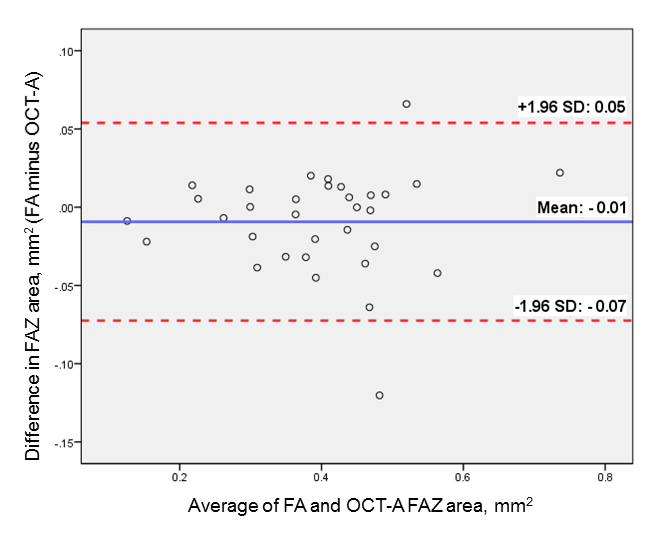


**Supplemental Table 1.** Univariate regression models of (a) foveal avascular zone (FAZ) area, (b) FAZ circularity, (c) total vessel density, (d) parafoveal vessel density and (e) fractal dimension and (f) vessel diameter index.

(a) FAZ area

| **Variables** | **Beta** | **95% CI** | **P value** |
| --- | --- | --- | --- |
| DR severity |  |  |  |
| Severe or above DR vs. No DR | 0.077 | 0.023 to 0.131 | **0.005** |
| Moderate DR vs. No DR | 0.050 | 0.012 to 0.087 | **0.010** |
| Mild DR vs. No DR | 0.047 | 0.008 to 0.085 | **0.017** |
| Presence of DME | 0.032 | -0.005 to 0.069 | 0.093 |
| LogMAR, per unit | 0.028 | -0.042 to 0.099 | 0.435 |
| Axial length, mm | -0.028 | -0.002 to -0.015 | **<0.001** |
| Anterior chamber depth, mm | -0.018 | -0.040 to 0.003 | 0.098 |
| Spherical equivalent, diopter | 0.008 | 0.002 to 0.014 | **0.006** |
| Central corneal thickness, µm | 7.45×10^-5^ | -4.09×10^-4^ to 5.57×10^-4^ | 0.763 |
| Intraocular pressure, mmHg | 0.002 | -0.004 to 0.007 | 0.479 |
| Central subfield macular thickness, µm | -0.001 | -0.001 to -3.08×10^-4^ | **0.003** |
| Average macular thickness, µm | -3.22×10^-4^ | -0.001 to 0.001 | 0.447 |
| Average GC-IPL thickness, µm | -1.63×10^-6^ | -0.001 to 0.001 | 0.988 |
| Average peripapillary RNFL thickness, µm | -4.02×10^-4^ | -0.002 to 0.002 | 0.681 |
| Subfoveal choroidal thickness, µm | 2.48×10^-4^ | 8.69×10^-5^ to 4.10×10^-4^ | **0.003** |
| Age, year | -4.68×10^-4^ | -0.002 to 0.001 | 0.541 |
| Gender (male vs. female) | -0.042 | -0.075 to -0.009 | **0.012** |
| Duration of diabetes, year | 0.001 | -0.001 to 0.002 | 0.432 |
| Fasting glucose, mmol/L | -0.003 | -0.011 to 0.005 | 0.429 |
| HbA1c, % | 0.007 | -0.003 to 0.018 | 0.170 |
| Total cholesterol, mmol/L | -0.008 | -0.027 to 0.012 | 0.441 |
| HDL cholesterol, mmol/L | 0.044 | -0.006 to 0.095 | 0.085 |
| LDL cholesterol, mmol/L | -0.008 | -0.035 to 0.019 | 0.568 |
| Creatinine, µmol/L | 1.56×10^-5^ | -3.11×10^-4^ to 3.14×10^-4^ | 0.925 |
| Estimated glomerular filtration rate, mL/min/1.73m^2^ | 6.23×10^-5^ | -6.35×10^-4^ to 7.59×10^-4^ | 0.861 |
| Systolic blood pressure, mmHg | -6.09×10^-5^ | -9.73×10^-4^ to 8.52×10^-4^ | 0.896 |
| Diastolic blood pressure, mmHg | -3.52×10^-4^ | -0.002 to 0.001 | 0.683 |
| Pulse pressure, mmHg | 4.06×10^-5^ | -0.001 to 0.001 | 0.933 |
| Body mass index, kg/m^2^ | -0.002 | -0.005 to 0.001 | 0.165 |
| Current smoking | 0.041 | -0.004 to 0.086 | 0.072 |
| Anti-hypertensive medication | -0.001 | -0.043 to 0.042 | 0.979 |
| Anti-dyslipidemic medication | -0.020 | -0.058 to 0.017 | 0.283 |
| History of heart disease | 0.014 | -0.022 to 0.051 | 0.430 |
| History of stroke | 0.013 | -0.038 to 0.064 | 0.612 |
| History of arrhythmia | 0.017 | -0.036 to 0.070 | 0.528 |
| History of renal disease | -0.004 | -0.050 to 0.042 | 0.857 |

**(b) FAZ circularity**

| **Variables** | **Beta** | **95% CI** | **P value** |
| --- | --- | --- | --- |
| DR severity |  |  |  |
| Severe or above DR vs. No DR | -0.073 | -0.126 to -0.019 | **0.008** |
| Moderate DR vs. No DR | -0.037 | -0.012 to -0.008 | **0.012** |
| Mild DR vs. No DR | -0.023 | -0.054 to 0.007 | 0.137 |
| Presence of DME | 0.010 | -0.027 to 0.046 | 0.603 |
| LogMAR, per unit | -0.109 | -0.182 to -0.037 | **0.003** |
| Axial length, mm | 0.001 | -0.009 to -0.001 | 0.860 |
| Anterior chamber depth, mm | -0.006 | -0.025 to 0.013 | 0.528 |
| Spherical equivalent, diopter | 0.003 | -0.002 to 0.008 | 0.263 |
| Central corneal thickness, µm | -1.81×10^-5^ | -4.09×10^-4^ to 5.57×10^-4^ | 0.763 |
| Intraocular pressure, mmHg | 0.001 | -0.003 to 0.006 | 0.599 |
| Central subfield macular thickness, µm | -0.001 | -0.001 to -2.90×10^-4^ | **<0.001** |
| Average macular thickness, µm | -8.96×10^-4^ | -0.002 to -2.59×10^-4^ | **0.006** |
| Average GC-IPL thickness, µm | 0.001 | -3.39×10^-4^ to 0.002 | 0.142 |
| Average peripapillary RNFL thickness, µm | 3.83×10^-4^ | -0.001 to 0.002 | 0.533 |
| Subfoveal choroidal thickness, µm | 1.21×10^-4^ | -4.83×10^-5^ to 2.91×10^-4^ | 0.162 |
| Age, year | -0.001 | -0.002 to 0.001 | 0.394 |
| Gender (male vs. female) | -0.024 | -0.048 to 4.34×10^-5^ | 0.050 |
| Duration of diabetes, year | -0.001 | -0.002 to 4.02×10^-4^ | 0.199 |
| Fasting glucose, mmol/L | -0.002 | -0.008 to 0.004 | 0.479 |
| HbA1c, % | -0.007 | -0.016 to 0.002 | 0.130 |
| Total cholesterol, mmol/L | 0.004 | -0.009 to 0.017 | 0.575 |
| HDL cholesterol, mmol/L | -0.001 | -0.031 to 0.030 | 0.962 |
| LDL cholesterol, mmol/L | 0.006 | -0.011 to 0.023 | 0.513 |
| Creatinine, µmol/L | -5.19×10^-4^ | -3.93×10^-4^ to 7.52×10^-4^ | 0.539 |
| Estimated glomerular filtration rate, mL/min/1.73m^2^ | 1.80×10^-5^ | -6.35×10^-4^ to 7.59×10^-4^ | 0.861 |
| Systolic blood pressure, mmHg | -0.001 | -0.001 to 3.05×10^-5^ | 0.062 |
| Diastolic blood pressure, mmHg | -4.00×10^-5^ | -0.001 to 0.001 | 0.946 |
| Pulse pressure, mmHg | -0.001 | -0.001 to 4.77×10^-5^ | 0.075 |
| Body mass index, kg/m^2^ | 0.001 | -0.002 to 0.004 | 0.467 |
| Current smoking | -0.021 | -0.063 to 0.020 | 0.315 |
| Anti-hypertensive medication | -0.008 | -0.037 to 0.021 | 0.574 |
| Anti-dyslipidemic medication | -0.001 | -0.028 to 0.026 | 0.917 |
| History of heart disease | 0.014 | -0.022 to 0.051 | 0.430 |
| History of stroke | -0.043 | -0.085 to -3.90×10^-5^ | **0.0498** |
| History of arrhythmia | 0.015 | -0.024 to 0.055 | 0.442 |
| History of renal disease | 0.007 | -0.049 to 0.034 | 0.726 |

**(c) Total Vessel Density**

| **Variables** | **Beta** | **95% CI** | **P value** |
| --- | --- | --- | --- |
| DR severity |  |  |  |
| Severe or above DR vs. No DR | -0.044 | -0.084 to -0.003 | **0.033** |
| Moderate DR vs. No DR | -0.045 | -0.063 to -0.027 | **<0.001** |
| Mild DR vs. No DR | -0.037 | -0.055 to -0.018 | **<0.001** |
| Presence of DME | -0.008 | -0.031 to 0.014 | 0.466 |
| LogMAR, per unit | -0.011 | -0.063 to -0.041 | 0.673 |
| Axial length, mm | -0.004 | -0.011 to 0.003 | 0.308 |
| Anterior chamber depth, mm | 0.009 | -0.003 to 0.021 | 0.125 |
| Spherical equivalent, diopter | -1.88×10^-4^ | -0.004 to 0.003 | 0.913 |
| Central corneal thickness, um | -1.43×10^-4^ | -4.30×10^-4^ to 1.42×10^-4^ | 0.324 |
| Intraocular pressure, mmHg | 1.35×10^-4^ | -0.003 to 0.003 | 0.923 |
| Central subfield macular thickness, µm | 8.77×10^-7^ | -2.17×10^-4^ to 2.18×10^-4^ | 0.994 |
| Average macular thickness, µm | 2.06×10^-4^ | -2.33×10^-4^ to 6.44×10^-4^ | 0.358 |
| Average GC-IPL thickness, µm | 0.001 | 2.48×10^-4^ to 0.002 | **0.010** |
| Average peripapillary RNFL thickness, µm | 0.001 | -1.08×10^-4^ to 0.002 | 0.086 |
| Subfoveal choroidal thickness, µm | -3.58×10^-5^ | -1.40×10^-4^ to 6.87×10^-5^ | 0.502 |
| Age, year | 0.001 | -6.00×10^-5^ to 0.002 | 0.070 |
| Gender (male vs. female) | -0.014 | -0.030 to 0.003 | 0.107 |
| Duration of diabetes, year | -1.04×10^-4^ | -0.001 to 0.001 | 0.818 |
| Fasting glucose, mmol/L | -0.003 | -0.008 to 0.001 | 0.164 |
| HbA1c, % | -0.007 | -0.015 to 2.44×10^-4^ | 0.058 |
| Total cholesterol, mmol/L | 0.011 | 0.002 to 0.020 | **0.022** |
| HDL cholesterol, mmol/L | 0.002 | -0.017 to 0.022 | 0.807 |
| LDL cholesterol, mmol/L | 0.017 | 0.005 to 0.029 | **0.005** |
| Creatinine, µmol/L | -5.10×10^-5^ | -1.84×10^-4^ to 8.17×10^-5^ | 0.958 |
| Estimated glomerular filtration rate, mL/min/1.73m^2^ | 1.02×10^-5^ | -3.74×10^-4^ to 3.95×10^-4^ | 0.451 |
| Systolic blood pressure, mmHg | 6.01×10^-5^ | -4.15×10^-4^ to 0.001 | 0.804 |
| Diastolic blood pressure, mmHg | 0.001 | -2.71×10^-4^ to 0.001 | 0.198 |
| Pulse pressure, mmHg | -1.01×10^-4^ | -0.001 to 3.78×10^-4^ | 0.679 |
| Body mass index, kg/m^2^ | 0.001 | -0.001 to 0.002 | 0.410 |
| Current smoking | -0.028 | -0.061 to 0.005 | 0.100 |
| Anti-hypertensive medication | 0.011 | -0.010 to 0.032 | 0.317 |
| Anti-dyslipidemic medication | 0.006 | -0.012 to 0.023 | 0.525 |
| History of heart disease | 0.002 | -0.018 to 0.022 | 0.845 |
| History of stroke | 0.003 | -0.027 to 0.033 | 0.856 |
| History of arrhythmia | 0.024 | -0.002 to 0.050 | 0.067 |
| History of renal disease | 0.019 | -0.005 to 0.042 | 0.127 |

**(d) Parafoveal vessel density**

| **Variables** | **Beta** | **95% CI** | **P value** |
| --- | --- | --- | --- |
| DR severity |  |  |  |
| Severe or above DR vs. No DR | -0.045 | -0.088 to -0.003 | **0.042** |
| Moderate DR vs. No DR | -0.045 | -0.065 to -0.027 | **<0.001** |
| Mild DR vs. No DR | -0.038 | -0.057 to -0.018 | **<0.001** |
| Presence of DME | -0.008 | -0.032 to 0.015 | 0.483 |
| LogMAR, per unit | -0.012 | -0.070 to 0.045 | 0.678 |
| Axial length, mm | -0.007 | -0.014 to 0.001 | 0.076 |
| Anterior chamber depth, mm | 0.008 | -0.005 to 0.021 | 0.234 |
| Spherical equivalent, diopter | 0.001 | -0.003 to 0.005 | 0.596 |
| Central corneal thickness, um | -1.32×10^-4^ | -4.41×10^-4^ to 1.77×10^-4^ | 0.403 |
| Intraocular pressure, mmHg | -2.32×10^-5^ | -0.003 to 0.003 | 0.988 |
| Central subfield macular thickness, µm | -8.03×10^-5^ | -3.10×10^-4^ to 4.19×10^-4^ | 0.492 |
| Average macular thickness, µm | 1.95×10^-4^ | -2.53×10^-4^ to 6.42×10^-4^ | 0.395 |
| Average GC-IPL thickness, µm | 0.001 | 3.81×10^-4^ to 0.002 | **0.004** |
| Average peripapillary RNFL thickness, µm | 0.001 | -5.30×10^-4^ to 0.002 | 0.065 |
| Subfoveal choroidal thickness, µm | -1.76×10^-5^ | -1.34×10^-4^ to 9.53×10^-5^ | 0.760 |
| Age, year | 0.001 | -1.07×10^-5^ to 0.002 | 0.053 |
| Gender (male vs. female) | -0.017 | -0.035 to 0.001 | 0.061 |
| Duration of diabetes, year | 2.43×10^-5^ | -0.001 to 0.001 | 0.960 |
| Fasting glucose, mmol/L | -0.004 | -0.009 to 0.001 | 0.092 |
| HbA1c, % | -0.007 | -0.015 to 0.001 | 0.076 |
| Total cholesterol, mmol/L | 0.010 | 3.35×10^-4^ to 0.020 | **0.043** |
| HDL cholesterol, mmol/L | 0.004 | -0.016 to 0.025 | 0.672 |
| LDL cholesterol, mmol/L | 0.017 | 0.004 to 0.030 | **0.012** |
| Creatinine, µmol/L | -5.27×10^-5^ | -1.86×10^-4^ to 8.02×10^-5^ | 0.437 |
| Estimated glomerular filtration rate, mL/min/1.73m^2^ | -5.41×10^-6^ | -4.15×10^-4^ to 4.04×10^-5^ | 0.979 |
| Systolic blood pressure, mmHg | 5.76×10^-5^ | -4.56×10^-4^ to 0.001 | 0.826 |
| Diastolic blood pressure, mmHg | 4.97×10^-4^ | -3.65×10^-4^ to 0.001 | 0.258 |
| Pulse pressure, mmHg | -9.60×10^-5^ | -0.001 to 4.18×10^-4^ | 0.715 |
| Body mass index, kg/m^2^ | 3.71×10^-4^ | -0.001 to 0.002 | 0.598 |
| Current smoking | -0.025 | -0.063 to 0.012 | 0.188 |
| Anti-hypertensive medication | 0.012 | -0.011 to 0.035 | 0.300 |
| Anti-dyslipidemic medication | 0.005 | -0.014 to 0.023 | 0.626 |
| History of heart disease | 0.006 | -0.015 to 0.027 | 0.582 |
| History of stroke | 0.004 | -0.028 to 0.035 | 0.825 |
| History of arrhythmia | 0.028 | 0.001 to 0.056 | **0.046** |
| History of renal disease | 0.019 | -0.006 to 0.043 | 0.138 |

**(e) Fractal dimension**

| **Variables** | **Beta** | **95% CI** | **P value** |
| --- | --- | --- | --- |
| DR severity |  |  |  |
| Severe or above DR vs. No DR | -0.011 | -0.017 to -0.004 | **0.001** |
| Moderate DR vs. No DR | -0.010 | -0.013 to -0.007 | **<0.001** |
| Mild DR vs. No DR | -0.008 | -0.011 to -0.005 | **<0.001** |
| Presence of DME | -0.003 | -0.006 to 4.34×10^-5^ | 0.054 |
| LogMAR, per unit | 0.004 | -0.005 to 0.013 | 0.395 |
| Axial length, mm | 0.001 | -3.75×10^-4^ to 0.002 | 0.168 |
| Anterior chamber depth, mm | 0.001 | -0.001 to 0.003 | 0.377 |
| Spherical equivalent, diopter | -0.001 | -0.001 to 9.37×10^-5^ | 0.098 |
| Central corneal thickness, um | -3.67×10^-5^ | -7.96×10^-5^ to 6.17×10^-6^ | 0.093 |
| Intraocular pressure, mmHg | 4.54×10^-6^ | -3.89×10^-4^ to 3.98×10^-4^ | 0.982 |
| Central subfield macular thickness, µm | 2.30×10^-6^ | -4.26×10^-5^ to 4.72×10^-5^ | 0.920 |
| Average macular thickness, µm | -3.24×10^-5^ | -1.26×10^-4^ to 6.08×10^-5^ | 0.496 |
| Average GC-IPL thickness, µm | 4.04×10^-6^ | -1.33×10^-4^ to 1.42×10^-4^ | 0.954 |
| Average peripapillary RNFL thickness, µm | -1.49×10^-6^ | -1.61×10^-4^ to 1.58×10^-4^ | 0.985 |
| Subfoveal choroidal thickness, µm | -2.00×10^-5^ | -3.67×10^-5^ to -3.24×10^-6^ | **0.019** |
| Age, year | 1.08×10^-4^ | -2.64×10^-5^ to 2.43×10^-4^ | 0.115 |
| Gender (male vs. female) | -0.002 | -0.004 to 0.001 | 0.295 |
| Duration of diabetes, year | -4.66×10^-5^ | -1.98×10^-4^ to 1.04×10^-5^ | 0.546 |
| Fasting glucose, mmol/L | -0.001 | -0.001 to 5.89×10^-5^ | 0.074 |
| HbA_1c_, % | -0.001 | -0.002 to -9.64×10^-5^ | **0.033** |
| Total cholesterol, mmol/L | 0.001 | -0.001 to 0.003 | 0.248 |
| HDL cholesterol, mmol/L | -4.19×10^-5^ | -0.002 to 0.003 | 0.811 |
| LDL cholesterol, mmol/L | 0.002 | -3.32×10^-4^ to 0.004 | 0.101 |
| Creatinine, µmol/L | -2.38×10^-6^ | -4.64×10^-5^ to 4.16×10^-5^ | 0.916 |
| Estimated glomerular filtration rate, mL/min/1.73m^2^ | -2.10×10^-5^ | -9.75×10^-5^ to 5.56×10^-5^ | 0.592 |
| Systolic blood pressure, mmHg | 2.54×10^-5^ | -5.98×10^-5^ to 1.11×10^-4^ | 0.559 |
| Diastolic blood pressure, mmHg | 9.86×10^-5^ | -4.14×10^-5^ to 2.39×10^-4^ | 0.168 |
| Pulse pressure, mmHg | -3.45×10^-6^ | -8.69×10^-5^ to 8.00×10^-4^ | 0.935 |
| Body mass index, kg/m^2^ | 1.63×10^-4^ | -6.01×10^-5^ to 3.86×10^-4^ | 0.152 |
| Current smoking | -0.005 | -0.010 to -0.001 | **0.019** |
| Anti-hypertensive medication | -0.001 | -0.004 to 0.003 | 0.655 |
| Anti-dyslipidemic medication | 2.88×10^-4^ | -0.003 to 0.003 | 0.852 |
| History of heart disease | -0.001 | -0.004 to 0.002 | 0.588 |
| History of stroke | -4.80×10^-4^ | -0.007 to 0.006 | 0.885 |
| History of arrhythmia | 0.003 | -0.002 to 0.007 | 0.264 |
| History of renal disease | 0.005 | 1.61×10^-5^ to 0.010 | **0.043** |

**(f) Vessel diameter index**

| **Variables** | **Beta** | **95% CI** | **P value** |
| --- | --- | --- | --- |
| DR severity |  |  |  |
| Severe or above DR vs. No DR | 6.06×10^-4^ | 1.69×10^-5^ to 0.001 | **0.007** |
| Moderate DR vs. No DR | 5.27×10^-4^ | 3.12×10^-4^ to 7.42×10^-4^ | **<0.001** |
| Mild DR vs. No DR | 5.20×10^-4^ | 2.51×10^-4^ to 7.89×10^-4^ | **<0.001** |
| Presence of DME | 1.15×10^-4^ | -1.45×10^-4^ to 3.76×10^-4^ | 0.386 |
| LogMAR, per unit | -2.15×10^-5^ | -8.32×10^-4^ to 4.03×10^-5^ | 0.496 |
| Axial length, mm | -8.67×10^-5^ | -1.81×10^-4^ to 8.32×10^-6^ | 0.074 |
| Anterior chamber depth, mm | -8.44×10^-6^ | -1.61×10^-4^ to 1.45×10^-4^ | 0.914 |
| Spherical equivalent, diopter | 3.19×10^-5^ | -8.73×10^-6^ to 7.25×10^-5^ | 0.124 |
| Central corneal thickness, um | 1.185×10^-6^ | -1.82×10^-6^ to 4.20×10^-6^ | 0.441 |
| Intraocular pressure, mmHg | -2.01×10^-7^ | -3.72×10^-5^ to 3.24×10^-5^ | 0.990 |
| Central subfield macular thickness, µm | 7.68×10^-7^ | -2.25×10^-5^ to 3.79×10^-5^ | 0.619 |
| Average macular thickness, µm | 2.89×10^-6^ | -2.82×10^-6^ to -8.60×10^-6^ | 0.292 |
| Average GC-IPL thickness, µm | 5.48×10^-5^ | -4.37×10^-6^ to 1.53×10^-5^ | 0.276 |
| Average peripapillary RNFL thickness, µm | 4.93×10^-6^ | -6.21×10^-6^ to 1.61×10^-5^ | 0.386 |
| Subfoveal choroidal thickness, µm | 9.32×10^-9^ | -1.14×10^-6^ to 1.83×10^-5^ | 0.987 |
| Age, year | 7.20×10^-6^ | -4.16×10^-6^ to 1.86×10^-5^ | 0.214 |
| Gender (male vs. female) | 4.51×10^-5^ | -1.71×10^-4^ to 2.61×10^-4^ | 0.682 |
| Duration of diabetes, year | 7.68×10^-6^ | -2.94×10^-6^ to 1.83×10^-5^ | 0.156 |
| Fasting glucose, mmol/L | 7.84×10^-5^ | 2.90×10^-5^ to 1.28×10^-4^ | **0.002** |
| HbA1c, % | 8.93×10^-5^ | 4.22×10^-6^ to 1.74×10^-4^ | **0.040** |
| Total cholesterol, mmol/L | -1.62×10^-5^ | -1.31×10^-4^ to 9.84×10^-5^ | 0.782 |
| HDL cholesterol, mmol/L | -3.81×10^-5^ | -2.98×10^-4^ to 2.22 ×10^-4^ | 0.774 |
| LDL cholesterol, mmol/L | -4.99×10^-5^ | -2.07×10^-4^ to 1.07×10^-4^ | 0.532 |
| Creatinine, µmol/L | -4.51×10^-7^ | -2.87×10^-6^ to 1.97×10^-6^ | 0.715 |
| Estimated glomerular filtration rate, mL/min/1.73m^2^ | 1.40×10^-7^ | -5.73×10^-6^ to 6.01 ×10^-6^ | 0.963 |
| Systolic blood pressure, mmHg | -6.68×10^-7^ | -6.42×10^-6^ to 5.09×10^-6^ | 0.820 |
| Diastolic blood pressure, mmHg | -1.04×10^-5^ | -2.03×10^-5^ to -4.74×10^-7^ | **0.040** |
| Pulse pressure, mmHg | 2.63×10^-6^ | -3.33×10^-6^ to 8.59×10^-6^ | 0.388 |
| Body mass index, kg/m^2^ | -8.98×10^-6^ | -2.85×10^-5^ to 1.05×10^-5^ | 0.366 |
| Current smoking | 1.29×10^-4^ | -2.50×10^-4^ to 5.08 ×10^-5^ | 0.504 |
| Anti-hypertensive medication | -1.12×10^-5^ | -2.68×10^-4^ to 3.41×10^-4^ | 0.932 |
| Anti-dyslipidemic medication | 9.61×10^-5^ | -1.49×10^-4^ to 3.41×10^-4^ | 0.442 |
| History of heart disease | 1.89×10^-4^ | -8.61×10^-5^ to 4.65×10^-5^ | 0.178 |
| History of stroke | 2.30×10^-5^ | -4.08×10^-4^ to 4.54×10^-4^ | 0.917 |
| History of arrhythmia | -7.95×10^-5^ | -4.39×10^-4^ to 2.80×10^-5^ | 0.665 |
| History of renal disease | -2.29×10^-4^ | -5.72×10^-4^ to 1.15×10^-4^ | 0.192 |

DR: diabetic retinopathy; DME: diabetic macular edema; FAZ: Foveal avascular zone; GC-IPL= ganglion cell inner plexiform layer LogMAR: logarithm of the minimum angle of resolution; HDL=high-density lipoprotein; LDL= Low-density lipoprotein; OCT-A: optical coherence tomography angiography; RNFL= retinal nerve fibre layer; SE: standard error.
